# Role of Infection and Immunity in Bovine Perinatal Mortality: Part 2. Fetomaternal Response to Infection and Novel Diagnostic Perspectives

**DOI:** 10.3390/ani11072102

**Published:** 2021-07-15

**Authors:** Paulina Jawor, John F. Mee, Tadeusz Stefaniak

**Affiliations:** 1 Department of Immunology, Pathophysiology and Veterinary Preventive Medicine, Wrocław University of Environmental and Life Sciences, 50-375 Wrocław, Poland; tadeusz.stefaniak@upwr.edu.pl; 2 Animal and Bioscience Research Department, Teagasc, Moorepark Research Centre, P61 P302 Fermoy, County Cork, Ireland; john.mee@teagasc.ie

**Keywords:** calf, intrauterine infection, fetal immune response, fetal inflammatory response syndrome, stillbirth/perinatal mortality

## Abstract

**Simple Summary:**

Bovine perinatal mortality (death of the fetus or calf before, during, or within 48 h of calving at full term (≥260 days) may be caused by noninfectious and infectious causes. Although infectious causes of fetal mortality are diagnosed less frequently, infection in utero may also compromise the development of the fetus without causing death. This review presents fetomaternal responses to infection and the changes which can be observed in such cases. Response to infection, especially the concentration of immunoglobulins and some acute-phase proteins, may be used for diagnostic purposes. Some changes in internal organs may also be used as an indicator of infection in utero. However, in all cases (except pathogen-specific antibody response) non-pathogen-specific responses do not aid in pathogen-specific diagnosis of the cause of calf death. But, nonspecific markers of in utero infection may allow us to assign the cause of fetal mortality to infection and thus increase our overall diagnosis rate, particularly in cases of the “unexplained stillbirth”.

**Abstract:**

Bovine perinatal mortality due to infection may result either from the direct effects of intrauterine infection and/or the fetal response to such infection, leading to the fetal inflammatory response syndrome (FIRS). Both intrauterine infection and FIRS, which causes multi-organ damage and involution of immune organs, compromise fetal survivability, sometimes fatally. Organ injury associated with FIRS may, in addition to causing fetal mortality, irreversibly compromise extrauterine adaptation of the neonate, a recognized problem in human fetuses. Diagnosis of intrauterine infection and of FIRS requires related, but independent analytical approaches. In addition to detection of pathogens, the immune and inflammatory responses of the bovine fetus may be utilized to diagnose intrauterine infection. This can be done by detection of specific changes in internal organs and the measurement of antibodies and/or elements of the acute phase reaction. Currently our ability to diagnose FIRS in bovine fetuses and neonates is limited to research studies. This review focuses on both the fetomaternal response to infection and diagnostic methods which rely on the response of the fetus to infection and inflammatory changes, as well other methods which may improve diagnosis of intrauterine infection in cases of bovine perinatal mortality.

## 1. Introduction

One of the many animal health problems which decrease dairy and suckler herd productivity is perinatal morality (PM). Perinatal mortality may be defined as death of the fetus or calf before, during or within 48 h of calving at full term (≥260 days) [1,2].

Large scale, multi-farm studies suggest that 2.4–9.7% of dairy calves die in the perinatal period across a wide range of different systems, with an unweighted mean of 6.2% [3]. However, the herd-level prevalence of perinatal calf mortality is positively skewed [4] and can vary between 0 and 30.6% in dairy herds [5]. Important risk factors for PM include age at first calving [6], breed of dam, breeding method, calving management, fetomaternal health status, length of gestation [7], gestational nutrition, calf sex, and sire [8]. While many risk factors for PM are not under management control (year of calving, month of calving, twin calving, primiparity, previous perinatal mortality, and fetal gender) [2], many others are modifiable [8].

Despite (or perhaps because of) the increasing productivity of dairy cows, the prevalence of PM in Holsteins has been systematically growing from the 1980s in many [1,9], but not all countries internationally [10]. This increase predominantly affects Holsteins. In contrast, in the Norwegian Red breed the prevalence of stillborn calves did not change from 1978 to 2004 [11].

In a recent study the effects of stillbirth on productive and reproductive performance as well as financial losses due to PM were evaluated [12]. The financial losses associated with stillbirth averaged US$ 938 per case (range from $US 767 to $US 1189 among nine farms).

## 2. Diagnosis of Infectious Stillbirth

The diagnosis of the causes of calf mortality is a challenge. Depending on the capabilities and experience of the diagnostician/s and the available laboratory support, and many other factors, the proportion of identified causes of death varies. Undiagnosed cases are a serious problem in investigating the causes of death of calves, as without knowing the cause it is impossible to prevent losses in future. In approximately one-third to a half of perinatal mortality cases, no cause of death can be detected [1,13,14].

When studying the causes of abortion or PM, infection is always more commonly detected in aborted than in stillborn fetuses [15,16,17]. In PM cases, signs of infection were detected in 10–21% of cases among different studies [16,18]. The low percentage of PM cases diagnosed with infection may be the result of the varying methodologies of different research studies. The fact that in a significant proportion of undiagnosed cases, signs of inflammation (e.g., encephalitis, hepatitis, myocarditis, placentitis, pneumonia, pleuritis, other) were found at necropsy [19] suggests that the occurrence of intrauterine infection in cattle is underestimated. Diagnosing an intrauterine infection following necropsy is less frequent where material for detailed diagnostic tests is collected only in cases when there are gross lesions and cases without gross lesions are not tested for infection [1,16]. In contrast, where all calves are examined for the presence of bacteria (samples cultured aerobically, anaerobically, and in microaerobic conditions), viruses (BVDV, BoHV-1, SBV), and *Neospora caninum*, pathogen-specific diagnosis rates can be higher [18]. In one recent study, approximately one in five PM calves were exposed to a pathogen during pregnancy [18]. Such an exhaustive investigation is not always possible due to the associated costs. The most recent review of the classical diagnostic approaches to infectious causes of PM is found in Mee et al. [20].

**Figure 1:**
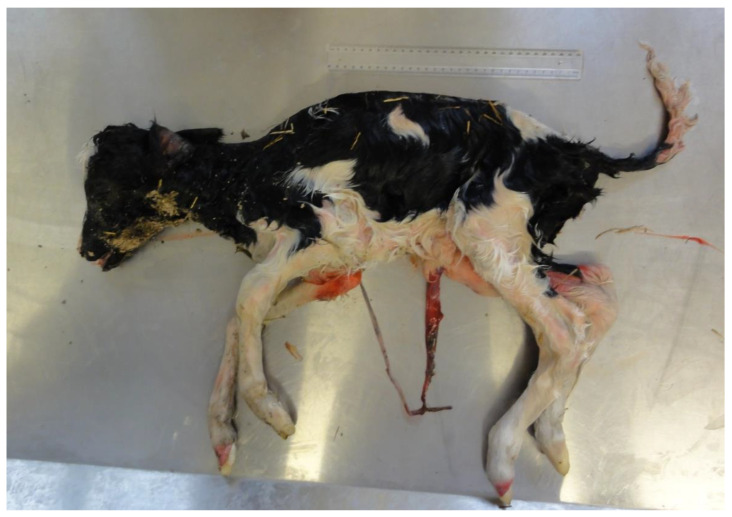
Intra-uterine growth retardation (IUGR) may be a sub-lethal or lethal consequence of an infection in utero (in this case, *Leptospira hardjo*, 261-day gestation, 7.8 kg). Note: ruler length 30 cm.

At its most simple, the role of infection in PM can be that it kills the fetus or perinate. However, sub-lethal infection may cause many other problems which either indirectly increases the risk of death (e.g., shortened gestation or intrauterine growth retardation (IUGR) (Figure 1) [18,21], or result in the birth of live, but non-viable, calves (e.g., weak calf syndrome due to adenovirus) [22] or live and viable, but transiently or persistently infected calves (e.g., infection with BVDV or *N. caninum*) [23,24]. Smyth et al. [25] found that cows with leptospiral antigens in the placenta had significantly lighter fetuses, by an average of 6 to 10 kg, than those from cows with no antigen in the placenta. Intrauterine growth retardation of calves that survive BVDV-infection on day 100 of gestation has also been reported [26]. Thus the effects of pre or perinatal infection may extend beyond the perinatal period.

Transmission routes of infection from the dam to the fetus include migration from the abdominal cavity through the fallopian tubes, hematogenous spread through the placenta, and passage through the cervix from the vagina [27]. In addition, for some bacteria *(Trueperella pyogenes*, and *Campylobacter fetus*), their persistence in utero prior to pregnancy may occur [28]. Kirkbride et al. [29] suggested that the hematogenous route seems to be the most important in bovines. Maternal immunosuppression during early pregnancy may be regulated through the down-expression of Th1 cytokines (IFN-γ and IL-2) and up-expression of Th2 cytokines, which leads to a successful pregnancy in cattle. Th1 cytokines are generally harmful to the maintenance of pregnancy, whereas the Th2 cytokines support the humoral response and successful pregnancy [30]. This inhibition of cell-mediated immune response may cause an increased risk of infection transmission from cow to the fetus. The efficient immune response against *N. caninum* requires a Th1 cytokine response involving IFN-γ to control parasite multiplication, although an excess of IFN-γ in the placenta may have detrimental effects for gestation and compromise fetal viability [31].

Infection can shorten the length of gestation and this may be a clue in the diagnosis of infection cases. For example, in a recent study in infected singletons, gestation length was shorter than in uninfected singletons (274 ± 8 vs. 279 ± 7 days; *p* < 0.01). The odds ratio for diagnosis of infection in single pregnancies <275 days was 3.75 (95% CI: 1.2–12.1), (*p* < 0.05) [18]. The shorted gestation is not the one negative consequence of an infection in utero. Fetal response and changes in internal organs are discussed below.

## 3. Development of Immune System in Bovine Fetuses and Response to Antigenic Stimulation in Utero

### 3.1. Development of the Cellular Components of the Bovine Fetal Immune System

The fetal immunological response develops throughout pregnancy. Germinal centers of immune organs appear at early stages of fetal development [32,33,34], e.g., germ of thymus is present at 42 day, spleen on 55 day of bovine fetal life). Phagocytic cells (granulocytes and monocytes) appear in the fetal circulating blood at approximately the 130th day of pregnancy [35,36]. Initially, these cells play only a limited role in fetal immunity. In the final stage of pregnancy, fetal neutrophil granulocytes are able to phagocytose, but their efficiency in killing the bacteria is low. In the perinatal period, the bactericidal ability of neutrophils is reduced because of the rise in cortisol concentration [37]. Bovine fetal lymphocytes appear in small numbers in the blood after the 40th day of pregnancy [35]. They respond to mitogens at the 14th week of pregnancy, whereas at the 24th week they respond with IFN-γ production upon the antigenic stimulation [38]. The occurrence of predominantly B lymphocyte subpopulations (IgM^+^) in follicles of bovine fetal ileal Peyers patches and T (CD3+ in the interfollicular area and the dome region) was confirmed in the 6th month of gestation [39]. These developments allow the fetus to recognize and respond to pathogens. The course of infection depends on the immune response [38] of the fetus as well as the virulence of the pathogen [40]. Fetal immunocompetence at the time of infection might play a key role in fetal survival, limiting lesions in fetal tissues [41].

#### Complement in the Fetus

The activity of complement has been detected in bovine fetal blood as early as the 75th day and at the 90th day reaches measurable levels [42]. Its activity remains 2–3 times lower than in adult cattle until the end of pregnancy [34,43].

### 3.2. Humoral Immune Response of the Bovine Fetus

The bovine fetus can produce IgM and IgG antibodies by the second trimester [44]. For example, the bovine fetus is able to produce antibodies against the PI-3 virus at 120 days, against BVDV at 190 days, and by the third trimester of pregnancy against the majority of antigens [36]. The presence of IgM in fetal serum has been detected at day 90 and IgG and IgA at day 111 of pregnancy [44]. In the study by Schultz et al. [35], the presence of IgM was confirmed at the earliest at 130 days and IgG at 145 days.

Bovine fetuses from 195 to 253 days gestational age had the capacity to mount cell-mediated and humoral responses of similar character and magnitude as adult cattle to tetanus toxoid [45]. Bovine fetuses immunized with coronavirus antigens 9–49 days before calving developed plasmocytes (IgG, IgM, and IgA positive) in the lamina propria of the ileum, in the lymphatic nodules and lymph nodes. These cells were absent in nonimmunized newborns, except for a few IgG-positive cells in the lamina propria of the ileum. In utero immunized calves were resistant to experimental challenge with homologous virus [46]. Bovine fetuses immunized orally by intraamniotic inoculation of killed *E. coli* O26 given 9–102 days before calving survived experimental challenge at birth with the same alive strain, although they were deprived of colostrum, whereas control calves died within 2–10 days. IgG and IgM antibodies were present in the jejunum and ileum, and IgG in jejunal lymph nodes. [47]. Bovine fetuses immunized with the same killed *E. coli* strain orally 10–50 days before birth expressed IgG and IgM containing cells in the jejunum, jejunal lymph node, and ileum. The high incidence of abortions after this route of vaccination in field conditions excluded the further development of this concept [48].

### 3.3. Fetal Inflammatory Response Syndrome (FIRS)

#### 3.3.1. FIRS in Humans Fetuses

The fetus, when exposed to microorganisms, or alternatively to noninfection-related stimuli (e.g., danger signals, or alarmins), can respond with local or systemic inflammatory actions. The term “fetal inflammatory response syndrome” (FIRS) was coined in human medicine in the late 90’s to describe a condition characterized by evidence of a systemic inflammatory response in human patients at risk for intra-amniotic infection who presented preterm labor with intact membranes or preterm prelabor rupture of the membranes [49]. FIRS in humans can be diagnosed by an increased concentration of umbilical cord plasma or serum acute phase reactants such as C-reactive protein or cytokines (e.g., interleukin-6) [50]. Pathologic evidence of a systemic fetal inflammatory response of human fetuses indicates the presence of funisitis and/or chorionic vasculitis [51]. However, FIRS can also be observed in patients with sterile intra-amniotic inflammation, alloimmunization (e.g., Rh disease), and active autoimmune disorders. An elevation in fetal plasma IL-6 can be observed in a subset of human fetuses with anemia due to Rh alloimmunization. This observation suggests that the hallmark of FIRS can be caused by noninfection-related insults. Further studies are required to determine whether the prognosis of FIRS caused by intra-amniotic infection/inflammation is different from that induced by alloimmunization [52]. High concentrations of multiple cytokines in cord blood, originating probably from fetal immune tissues, were detected in human neonates born to mothers with systemic autoimmune diseases [53]. This means that relying on just one indicator to diagnose FIRS may underdiagnose its occurrence. Human neonates born with FIRS have a higher rate of complications [50]. Although typically diagnosed in preterm human fetuses, FIRS can occur in term fetuses [54,55]. In FIRS, an influx of leukocytes and the synthesis of proinflammatory cytokines, including interleukins (IL-1α, IL-1β, IL-6, IL-8), occurs [56]. Interleukins are produced during tissue damage [57]. FIRS in humans is associated with multiorgan injury of the fetal hematopoietic system, thymus, adrenal glands, skin, kidneys, heart, lung, and brain [58,59]. It is speculated that the release of cytokines and other vasoactive substances in human intrauterine infection may cause vasospasm and alter blood flow to the fetus [60]. The FIRS may be present without clinical signs of inflammation. For example, in 12.8% of premature human births with no gross placental pathology, the following bacteria were isolated from amniotic fluid *Ureaplasma urealyticum, Fusobacterium* spp., and *Mycoplasma hominis* [54], and gestation was shorter, probably due to these infections. In humans, FIRS is evaluated as a risk factor for short- and long-term complications in babies (i.e., neonatal sepsis, bronchopulmonary dysplasia, periventricular leukomalacia, and cerebral palsy) [59]. It seems that systemic inflammation of the fetus, independent of the cause, is the main reason for adverse outcome caused by intrauterine infection. FIRS may limit the ability of the human fetus to adapt to extrauterine life and increase susceptibility to neonatal infections.

#### 3.3.2. FIRS in Animal Fetuses

Unlike in humans, there are very few reports of FIRS in bovine fetuses. This is not to imply that it does not occur, rather it reflects the difficulty in characterizing the bovine fetal inflammatory response. In fact much of the basic research on FIRS in humans was conducted in animal models, often using ovine fetuses [61,62].

As in humans, interleukin-6 should be a good biomarker of inflammation in the bovine fetus also. However, increased plasma concentrations of fetal IL-6 were not detected in PM calves with infection [63]. Moreover, IL-6 concentrations in plasma and in abomasum fluid were higher in living compared to dead calves [63]. The probable reason for this was that all the dead calves in that study were infected early in pregnancy, not at calving, since only calves with fetal antibodies were examined. Similarly, a stillborn calf with *Salmonella* Stanley infection, did not show increased IL-6 in serum [64]. However, it cannot be ruled out that increased levels may occur in acutely infected cases. The major problem with this marker is its short half-life. The half-time of elimination of recombinant human IL-6 lasts from minutes to hours (in rats [65]), which obviously considerably limits the usefulness of this biomarker in stillborn calves, if the half-time in bovines is similar.

Fetal inflammation is the main cause of subsequent adverse outcomes, not only for human neonates. In fetal piglets, experimental infection with porcine reproductive and respiratory syndrome virus (PRRSV) caused increased susceptibility to *Streptococcus suis* challenge during the neonatal period [66]. The authors suggested that the effects of PRRSV on the immune system may have cause increased susceptibility to bacterial infections after birth. In the case of Gram-negative bacterial infections, fetal loss may result, in part, from host responses to the bacterial cell wall component, lipopolysaccharide (LPS). LPS has been known to cause fetal death or abortion in animals since the 1940s [67] and still is frequently used in animal models to evaluate the response of the fetus to infection [61,68]. Intra-amniotic injection of LPS in piglets induced gut and lung immune responses and postnatal systemic inflammation in preterm piglets [68]. After delivery by caesarian section on the 103rd day of pregnancy (preterm), LPS-treated piglets showed a higher incidence of splenic bacterial accumulation (especially *Staphylococcus*) compared to a control group that received saline or no injection [68].

In humans, the organs most frequently affected by the consequences of FIRS are the brain, lungs, and thymus [50]. Studies from animal sciences show that after intrauterine inflammation/infection, these changes are not unique to human fetuses.

### 3.4. Brain

LPS is used in animal models to evaluate the fetal response to infection due to its ability to induce expression of proinflammatory cytokines [50]. In ovine fetuses, experimental intra-amniotic administration of LPS caused a rise in astrocyte count in the brain and cerebellum after 14 days, but a decrease of oligodendrocytes in the white matter of the cerebellar cortex, caudate nucleus, and hypothalamus [62]. Neuron count in the brain cortex, hypothalamus, and substantia nigra was reduced [62]. The development of FIRS in mouse fetuses causes fetal neuronal injury in utero [69]. In the non-bovine species, fetus development of FIRS may allow the inflammatory mediators like IL-1β, TNF-α, IFN-γ, PGE_2_ to bypass the blood-brain-barrier (BBB) and cause fetal brain injury with alterations in cytokine expression, neuronal injury, and microglial activation [69,70,71]. The proinflammatory cytokines probably cross the BBB [71] and initiate a neuroinflammatory response in the mouse model [69]. The microglia are the resident macrophages of the CNS [72]. The process of neural tissue maturation in the mouse is associated with widespread apoptotic cell death of both neurons and glia, and the embryonic and postnatal microglia are involved in the phagocytosis of these apoptotic bodies [73]. The intraperitoneal injection in mice with *Salmonella* Typhimurium leads to activation of the cerebral endothelium and microglia [74].

Changes similar to human FIRS were detected in a case of bovine fetal infection with *S.* Stanley by histopathological examination of the brain [64]. Inflammatory cell infiltration of leptomeningeal vessels, focal gliosis, and necrotic foci with microglia cells were found. However, other changes like 50–250 mL of transudate in the pleural and peritoneal cavities, and brain foci of colliquative necrosis, which were detected, may also be associated with hypoxia and brain ischemia, followed by regenerative processes. Therefore, it cannot be excluded that hypoxia and brain ischemia occurred at the same time as intrauterine infection, and the detected changes were caused by both processes.

Due to the limited number of studies in which the effects of general infection on the brain were presented, results from studies describing brain inflammatory changes and infection, as well as its effect on fetal survivability, were analyzed. In cases of BVDV infection, where the virus has a tropism for the brain in bovine fetuses, changes depend on the time of infection. The nervous tissue is a primary target for persistent infection [75]. Definitive brain lesions (pseudocysts in the subependymal zone in the region of the median eminence and adjacent corona radiata as well as in the region of the external capsule associated with lenticulostriate arteriae) and BVDV antigens have been demonstrated in 190-day bovine fetuses when they were infected on 75 days of gestation, but not in acute, transiently infected fetuses inoculated on 175 days of gestation [75]. In all persistently infected fetuses, additional changes occurred in white matter areas at the tips of cerebrocortical gyri, in areas of the confluence of the corpus callosum–internal capsule as well as internal-external-extreme capsules, and/or in the white matter surrounding the lenticulostriate areas [75]. In the study of Ohmann [76], experimental infection with the BVDV virus in fetuses (120–165 days of pregnancy) affected the cerebellum in three fetuses (out of 4). Gross lesions were not observed, but a restricted, focal, or almost diffuse necrosis and depletion of cells in the external germinal layer, infiltration of mononuclear cells in leptomeninges and degeneration and depletion of Purkinje cells in zones corresponding to the altered external germinal layer were detected. BVDV-antigen was detected in the external germinal layer in the cerebellum of the three fetuses. The changes in the central nervous system after BVDV infection may be present in live born calves. Fetuses which survive BVDV infection are frequently born with neurological signs (e.g., ataxia, nystagmus, tremor, but may show no gross abnormalities (5 out of 6), and only microscopic changes (myelin deficiency, and presence abnormal glial cells) [77].

Experimental infection of *N. caninum* in early and late gestation resulted in substantial differences in the extent and pathological effects of infection [41]. In early gestation, multifocal to coalescing necrosis was observed in numerous fetal tissues, while in late gestation, changes in the fetuses were restricted to an occasional mild focal encephalitis and myelitis with the presence of parasite antigen in glial cells. There was no evidence of inflammatory infiltrates associated with tissue necrosis in any of the fetal tissues at day 70 of gestation, whereas a mononuclear cell infiltrate was associated with areas of parasite-associated necrosis in fetal tissues at 210 days of gestation.

Infection of bovine fetuses on 118 days of pregnancy with tachyzoites of *N. caninum* resulted (17 days later) in multifocal vascular-orientated microgliosis and necrotic foci surrounded by microglial cells in one fetal brain [78]. Additionally, small focal infiltrates of large mononuclear cells, sometimes associated with focal cellular necrosis, were present in the spinal cord. Small numbers of lymphocytes and plasma cells were present around vessels or within the meninges. Focal mixed mononuclear cell infiltrates (macrophages, lymphocytes, and rare plasma cells) were also present in the liver (primarily portal in location), renal cortices, lung, heart, and skeletal muscle [78]. Brain lesions are less prominent in the 3–4 month-old fetuses comparing to older fetuses infected at 7–8 months of pregnancy with *N. caninum* had multifocal nonsuppurative encephalitis and focal gliosis [79].

**Figure 2:**
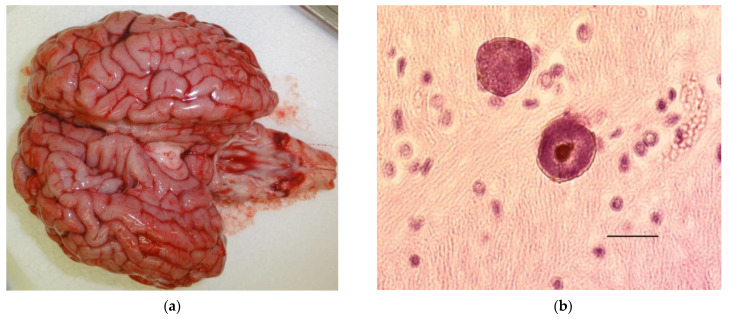
(**a**) (**left**) Grossly normal brain from a calf that died in the perinatal period with *Neospora caninum* cysts in its brain. (**b**) (**right**) *Neospora caninum* cysts in the brain of a calf that died in the perinatal period. Bar 20 µm.

These findings emphasize that microscopic evaluation of the fetal brain may be necessary as during necropsy, no changes may be visible see Figure 2a,b.

### 3.5. Lungs

A systemic inflammatory response during chorioamnionitis, is a risk factor for bronchopulmonary dysplasia in human fetuses [80]. The inflammatory response in human neonates is characterized by a rapid increase in inflammatory cells and of injurious mediators that can directly affect the alveolar-capillary unit and tissue integrity. An imbalance between pro-and anti-inflammatory cytokine influences causes activation of the cellular death pathways in the lungs, which is followed by healing (resolution of injury to a normal lung architecture) or repair. The disturbed alveolarization and angiogenesis during the repair after inflammation cause the development of bronchopulmonary dysplasia [81].

In animal sciences, fetal sheep receiving LPS for 28 days during mid-gestation developed signs of mild persistent inflammation in the fetal lung [82]. The etiology, time point, and duration of infection have an impact on pulmonary inflammation and induction of lung maturation [82]. In experimental conditions, intra-amniotic administration of *E.coli*-derived LPS induced IL-1-mediated lung inflammation and injury in sheep fetuses [83].

Although the lungs do not play an essential role during pregnancy, lung inflammation can adversely affect the calf after delivery. The available studies are focused only on the presence of inflammatory cells and hemorrhages in the lungs. Pneumonia was reported in 2% of stillborn beef calves at gross postmortem examination and in 5.8% following histological examination in Canada [15]. Severe pneumonia was described in one of 6 IUGR stillborn calves [84]. In a stillborn calf, focal and diffuse inflammatory infiltration and hemorrhages were found in the lungs, and significant growth of *Salmonella* Stanley was found in the culture from lungs and amniotic fluid [64]. Pneumonia was more commonly detected in stillborn calves with an abnormal thyroid gland compared to calves with a normal gland [85]. In fetal calves infected with the BVDV virus in mid-pregnancy, a marked peribronchiolar, lymphonodular hyperplasia was observed in the lungs [76]. Out of 8962 bovine fetuses in 565 stillborn and aborted calves without an isolated agent, the pneumonia was diagnosed by histopathology [19].

Although pneumonia in stillborn calves is an obvious sign of infection, there are no studies focusing on how infection affects the maturation of the lungs and hence how it influences the adaptation of calves after birth and their ability to survive. Anatomical maturity in cattle is achieved approximately 6 weeks before birth [86]. The extent to which such maturity is matched by functional maturity is unknown; however, it is recognized that significant development continues postnatally [87].

It is not known how an infection in bovine fetus will disturb this process. Considering the above data, the lungs warrant more careful examination in stillborn calves. Even in the absence of isolation of infectious agent, the nature of the lesions found histologically may indicate that the calf experienced intrauterine infection. The fetal lung inflammatory process seems to play a major role in the development of lung pathology and disturbed lung development and may influence the prognosis for the quality of life after birth in survivors.

### 3.6. Thymus

Intra-amniotic LPS administration to ovine fetuses at 106–113 days of pregnancy induced involution and activation of the fetal thymus [88]. In a further study, experimental intra-amniotic challenge of the fetal lambs with LPS resulted in decreased blood lymphocytes within 5 h and decreased thymic corticomedullary ratio within 24 h [89]. The mRNA for IL-1 and IL-6 was increased in the thymus. The authors concluded that a proinflammatory response associated with LPS exposure caused involution and persistent depletion of thymic Foxp3^+^ cells indicating a disturbance of fetal immune homeostasis. In the study of Kunzmann et al. [90] chorioamnionitis in fetal sheep induced by intraamniotic LPS injection led to involution of the fetal thymus, as indicated by activation of NF-κB in the thymus, the reduced thymic weight, but increased the number of circulating lymphocytes and of FoxP3+ positive thymocytes. The authors concluded that acute thymic involution, that was partly reversed after 5 days, may be in part the result of lymphocyte migration into the blood and inflamed organs such as the lung. It is suggested that FIRS influenced T_reg_ development and the immune system may have the plasticity to recover from stress and/or inflammation in utero.

However, thymic involution is not specific only for infection as in human fetuses with IUGR it may be a part of the fetal neuroendocrine response to intrauterine starvation [91].

After birth, the thymus has immune, regulatory and humoral functions [92]. Thymic atrophy was found in 6.1% of stillborn beef calves in Canada [15]. In Japanese Black calves that died with stillbirth/perinatal weak calf syndrome hypoplasia of lymphatic organs, especially the thymus, was observed, but no proinflammatory parameters were not examined in this study [93]. In one out of 6 fetuses born after natural infection with BVDV, a severe cortical hypoplasia of the thymus with a relative abundance of inter-lobular connective tissue was observed [77]. After infection of bovine fetuses at 120–165 days of gestation with BVDV, three out of four fetuses showed morphological immaturity without any pathological alterations [76]. Only in one fetus did necrosis and depletion of lymphocytes and infiltration of macrophages lead to hypoplasia [76]. Despite the ability of transiently infected fetuses to clear the virus, BVDV fetal infection during days 175–190 of gestation occurs during a critical stage of thymus and spleen development, as well as T cell selection and maturation in the thymus. BVDV fetal infection during this stage of fetal development may alter the animal’s ability to fight other infections postnatally [94].

Undergo of infection obviously influence the thymus gland, therefore, the examination may be an important element helping to diagnose intrauterine infection, although it should be performed with caution, as not all changes are specific.

**Figure 3:**
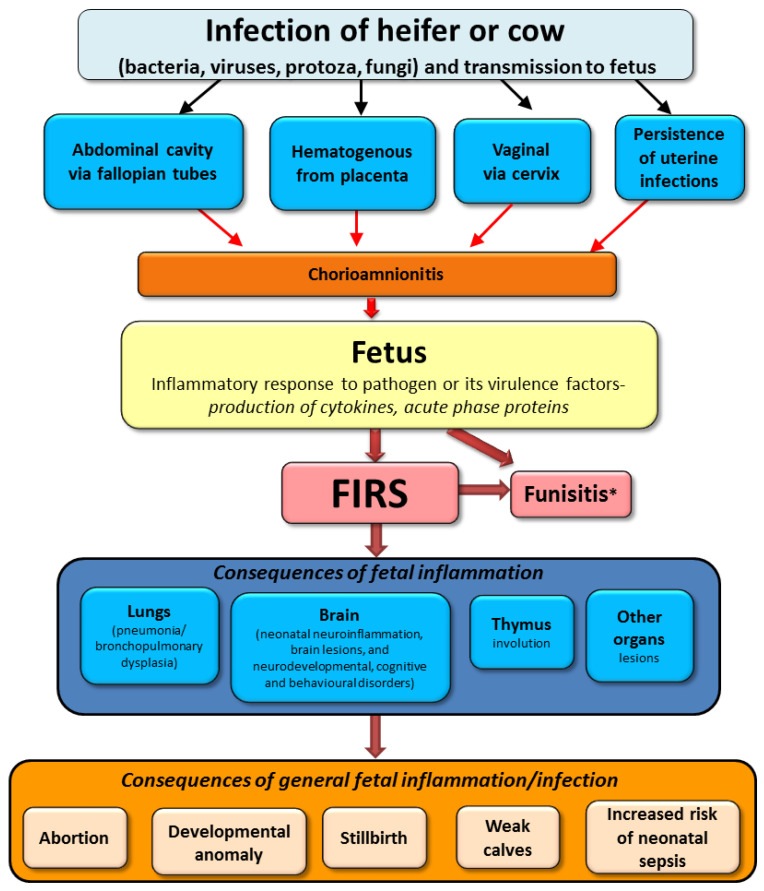
Possible sequelae of intrauterine infection in cattle based on human and animal studies (FIRS; fetal inflammatory response syndrome). * Funisitis-inflammation of umbilical vessels is also a hallmark of human FIRS.

In conclusion of above-described results from human and animal medicine, the possible course of infection in utero on fetal calf fate is presented in Figure 3.

## 4. Novel Approaches to Diagnosis Causes of Stillbirth

### 4.1. Monitoring of Fetus Responses

#### 4.1.1. Fetal Humoral Immune Response

The synepitheliochorial bovine placenta prevents the passage of immunoglobulins (Igs) from dam to fetus [95]. Although some studies [96,97] suggested possible leakage of maternal antibodies to the fetus, the probability of endogenous fetal antibody production, as detailed above, is higher. Hence, the antibodies detected in stillborn calves are likely to have originated from the fetal humoral immune response to infection. Fetal cells and deoxyribonucleic acid (DNA) have been detected in bovine maternal circulation, indicating transplacental leakage is possible, despite the synepitheliochorial placentation [98].

Due to the limitations in detecting infectious agents in calf carcasses, instead of attempting to detect infectious agents, it may be possible to use biomarkers of infection to explain some of the currently unexplained mortality. As detailed above, Igs from the dam do not cross the placenta and fetuses have the ability to produce Igs from the beginning of the second trimester [44]. Therefore, evaluation of Igs concentration in precolostral serum is one of the methods of detecting infection in utero, as many stillborn, premature or aborted fetuses have high Igs concentration in cases where the infection was detected [45,95].

Bovine fetuses infected with BVDV and *Histophilus somni*, had about 15-times higher IgM concentration and about 5-times higher IgG concentration in their precolostral serum than in the serum of uninfected newborns [99]. In bovine fetuses infected experimentally with *Neospora caninum,* IgG_1_ antibodies were detected in fluids of fetuses that succumbed, starting at 34 days postinfection (dpi), (104 days of gestation) and IgG_2_ from 41 dpi (111 days of gestation) [31]. Infected animals showed significant increases in their cytokine mRNA levels (IFN-γ, IL-4, IL-10, IL-12p40, and TNF-α) in the caruncle at the time of fetal death [31]. Nevertheless, the immune response of fetuses infected in early pregnancy was not protective against fetal mortality.

The classes of Igs which are detected depend on the type and time of infection. In bacterial infection (detected at full term) in calves, IgM concentration in the precolostral sera was higher compared to other causes of death [100]. In PM cases, where infections (parasitic, viral, or bacterial) were diagnosed by the specific Ig in calves blood, concentrations of IgG_1_ and IgG_2_, but not IgM, were significantly higher than in other causes of PM [63].

Detection of antibodies against specific pathogens is an important diagnostic parameter in the confirmation of intrauterine infection [18,100,101]. A recent study [18] showed that approximately 21% of PM calves in Polish dairy herds were exposed to pathogens during pregnancy; 15% had pathogen-specific antibodies, and 9% had pathogenic antigens detected (some had both). The higher prevalence of PM cases with pathogen-specific antibodies than pathogens could be due to the time lapse between infection and fetal death, sample collection issues, or limitations of the diagnostic methods, which are not as sensitive for autolytic samples. Therefore it seems that focusing on the antibody response gives a higher chance to detect contact with pathogens during pregnancy.

#### 4.1.2. Fetal Acute Phase Response

Apart from Igs, measurement of acute-phase proteins (APPs) like serum amyloid A (SAA) or haptoglobin (Hp) may be promising in detecting an acute inflammatory response in stillborn calves. The acute-phase response and production of APPs is nonspecific and it develops after any disturbance of homeostasis, but particularly inflammation [57]. In cattle, testing for APPs allows early and precise detection of inflammation. Recently upregulation of the immuno-inflammatory response in stillborn calves exposed to bacterial infection in utero has been demonstrated [100]. This research showed that SAA seems to be a better marker than Hp in the diagnosis of bacterial infections in stillborn calves [100]. In cases of bacterial infection, high APPs as a response to infection in stillborn calves may be similar to that described as FIRS in human fetuses. When using nonspecific biomarkers like APP, it should be remembered that other conditions may influence the results, and if calves live several hours before sampling (after birth), the SAA concentration may be increased due to calving [63].

#### 4.1.3. Fetal Metabolic Response-Perspectives

In the future new methods like metabolomics, which investigate metabolites that represent the functioning of an organism, should help in detecting calves infected in utero based on the specific metabolic profile of a calf or even the dam. Only one paper has been published describing different metabolite profiles in perinatal mortality cases [102], but it investigated only metabolic profiles in calves with different times of death, irrespective of the cause. A recent study showed promising results using metabolic profiles in facilitating diagnosis and treatment of calves with diarrhea [103], but the number of animals was small and this work needs to be reproduced in a larger population. Also, in cases of bovine respiratory diseases, metabolic profiles could be used to identify sick animals correctly in the majority of cases [104].

### 4.2. Monitoring of Dam Nonspecific Responses

Another approach to detecting the probability of PM, irrespective of causes, is focusing on the dam, although less chance of success is guaranteed in this approach. One study showed that in late gestation, high circulating neutrophil counts (higher than 1950/mm^3^) in heifers were associated with a lower risk of stillbirth [6], but other results did not support this finding [105]. In some heifers delivering stillbirths, aberrant profiles of oestrone sulphate and/or pregnancy-associated glycoproteins may be found [105]. While investigation of the usefulness of dam endocrine profiles in fetal well-being have been made, the number of stillborn calves was too small to obtain a clear picture [106]. It seems that changes in the hormones of dams may be useful in identifying stillborn cases. In the last 2 weeks of pregnancy and on the day of parturition, heifers with stillborn calves had a lower estradiol-17β concentration, whereas the prepartum progesterone concentration was greater (d 15 to 11 prepartum) than in heifers with live calves [107]. Unlike in fetuses, determination of Hp or SAA in dams failed to correlate with infection in fetuses [63], probably due to the chronicity of infection (it occurred before, not at, calving, as calves had time to mount an antibody response). Moreover, the physiologically relatively high concentration of acute-phase proteins in dams at calving may mask any possible maternal APP response to infection [63,108].

Studies in human medicine have shown that the determination of fetal fibronectin (FN) may be helpful with detecting the risk of intra-amniotic infection/inflammation and preterm delivery [109]. In women, plasma FN concentration in puerperal women was higher than in nonpregnant individuals [110]. Therefore it was hypothesized that the changes in FN concentration and occurrence of FN-fibrin complexes in maternal plasma might be associated with the perinatal mortality of calves. In cases where bovine fetuses die in utero and autolysis is evident at necropsy, dams show lower concentrations of fibronectin and supramolecular fibronectin–fibrin complexes compared to dams which delivered a live calf, but not to dams that delivered a nonautolyzed stillborn calf (the fetuses were not divided according to infectious/noninfectious group) [111]. This might have been associated with periparturient changes in tissues, as the first group died in utero [111]. In the same study, the cows which delivered a stillborn calf had circulating soluble FN-fibrin complexes with molecular masses from 1300 kDa to 1900 kDa. Control dams that delivered healthy calves had complexes of 750 kDa to 1000 kDa. In humans circulating soluble FN-fibrin complexes with molecular masses from around 750 kDa to 2100 kDa were present in puerperal plasma samples in patients with some diseases, especially when inflammation was present, but rarely in healthy individuals [112,113,114]. For these changes detected in dams to be used as a marker of stillbirth in cattle requires further research. Little is known about FN in cattle, but it is time to find new diagnostic tools that may increase our ability to prevent perinatal mortality and not only describe its causes.

The overlapping of different maternal events and their influence on the final response appear too complicated to have usefulness in diagnosing calf infections.

## 5. Conclusions

The reviewed results from different studies suggest that the consequences of inflammatory response syndrome in the fetus may in calves (as it was proved in humans, mice, rats, and sheep) cause irreversible injury of organs including the brain, lungs, and thymus. It may reduce the neonate’s ability to adapt to extrauterine life and limit their survival. Moreover, the increased susceptibility to neonatal infections in survivors of FIRS may be associated with organ failure and inhibited immune reactivity. The development of new diagnostic methods is necessary in order to detect with higher precision cases of intrauterine infection in bovine PM. Therefore, the highest probability of detection of infection in PM cases would be by not only searching for direct evidence of infection (pathogen detection) but also evaluating the presence of indirect indications (elevated Igs and APPs concentration in the precolostral plasma) as well as internal organ microscopic evaluation. However, some of these modalities may not always be possible due to cost.

Although there is no solid proof that FIRS exist in the same form in bovine fetuses as in human medicine and increased IL-6 concertation in bovine fetus are not proven yet, results which consist of bovine fetal responses (acute phase proteins and immunoglobulins) and changes in inner organs suggest that these phenomena are present here as well. A better understanding of FIRS in bovines and new diagnostic modalities may help in the evaluation of infectious causes of SB in cattle.

The significant economic losses associated with perinatal pathology prompt to formulate questions, e.g., are we able to detect the risk of weak calf syndrome development during pregnancy, or are we able to prevent its development in diagnosed cases? Progress obtained in this area in human perinatology gives the hope that the answer may be yes, but further study is required.

## Data Availability

No new data were created or analyzed in this study. Data sharing is not applicable to this article.
